# Retraction: Integrative analyses identify osteopontin, LAMB3 and ITGB1 as critical pro-metastatic genes for lung cancer

**DOI:** 10.1371/journal.pone.0320171

**Published:** 2025-03-04

**Authors:** The PLOS One Editors

After this article [1] was published, concerns were raised regarding the results presented in Fig 3 and Fig S6, the reported primer sequences, and potential cell line contamination.

Specifically:

- The following panels appear to partially overlap, despite representing different experimental conditions:
  ◦ Fig 3C MOCK and NC in the siRNA-LAMB3 results, Fig 3D MOCK and NC in the siRNA-OPN results, and Fig 3D MOCK and NC in the siRNA-LAMB3 results
  ◦ Fig 3D siRNA-OPN and Fig 3D siRNA-LAMB3
- In Figure S6B, there appears to be a vertical discontinuity between lanes 7 and 8 in the ITGB1 panel
- BLASTn analysis [2] of the siRNA sequences reported in this article [1] suggests that the siRNA sequences reported for LAMB3 and ITGB1 appear to target Osteopontin D.
- Following the publication of this article [1], the SPC-A-1 cell line reported in [1] was identified as a contaminated cell line and is a potential derivative of HeLa [3].

The first author commented that errors may have been made during the preparation of the Fig 3 results. They provided replacement images, but in the absence of the original triplicate image data used for quantification and the individual-level data underlying the graphs presented in Fig 3, these concerns cannot be fully resolved. Regarding Figure S6B, the first author states the original data are no longer available.

Regarding the cell line contamination concerns, the first author stated cell line authentication data from the time of the original experiments are not available. In the absence of this, the cell line contamination concerns cannot be resolved and the relevance of the reported results to lung cancer is in question.

In light of the above unresolved concerns, which question the integrity and reliability of the reported results and conclusions, the *PLOS One* Editors retract this article.

XMW did not respond to the final editorial decision. JL, MXY, LL, DSJ, QG, HCL, XHH, JJL, and MY either did not respond directly or could not be reached.
